# A 10-week physical therapist-supervised exercise program for nursing home residents with dementia: a single arm, observational feasibility study

**DOI:** 10.1016/j.jarlif.2025.100043

**Published:** 2025-11-03

**Authors:** Dennis Boer, Bente Winkler, Charlotte Schmidt, Shanty Sterke, Wilco Achterberg, Thea Vliet Vlieland

**Affiliations:** a Department of Strategy and Innovation, Kennemerhart, Haarlem, The Netherlands; b Department of Physiotherapy, University of Applied Sciences Leiden, Leiden, The Netherlands; c Department of Orthopedics, Rehabilitation and Physiotherapy, Leiden University Medical Center, Leiden, The Netherlands; d Research Centre Innovations in Care, Rotterdam University of Applied Sciences, Rotterdam, The Netherlands; e Department of Physiotherapy, Aafje Nursing Homes, Rotterdam, The Netherlands; f Department of Public Health and Primary Care, Leiden University Medical Center, Leiden, The Netherlands

**Keywords:** Exercise, Dementia, Alzheimer’s disease, Long-term care

## Abstract

**Background:**

Evidence on the effectiveness of exercise interventions for nursing home residence with dementia is scarce, with considerable practice variation with respect to their contents and dosage. This study aimed to evaluate the feasibility of an adequately dosed, personalized exercise intervention with respect to the assessment instruments, participants’ adherence and the occurrence of serious adverse events (primary feasibility outcomes) as well as the participant recruitment and participants’ and supervisors’ perceptions and experiences (secondary feasibility outcomes).

**Design:**

Single-arm observational study.

**Setting:**

Two nursing homes in Haarlem, the Netherlands.

**Participants:**

Nursing home residents with a confirmed diagnosis of dementia who were able to walk 50 meters (with or without walking aid), without expected resistance to the intervention.

**Intervention:**

10-week program, with two group-based sessions including strength and balance exercises, and two individual exergaming cycling sessions per week. The sessions were tailored to the participant via standardized assessments and supervised by a physical therapist.

**Results:**

Of 59 residents screened, 11 enrolled. Four of six clinical assessments were completed by all, and two by nine and ten participants, respectively. Nine participants completed both components, one only the individual exergaming part, and one participant dropped out. Adherence rates were 92 % for the group and 87 % for the individual sessions. Among 137 reported adverse events, nine were possibly related to the intervention, all minor and transient. The median participant appraisal score was 4.3 (out of five). Supervisors highlighted dementia-specific knowledge, individualized communication, and tailored approaches as facilitators, while scheduling conflicts posed challenges.

**Conclusion:**

A 10-week, personalized, physical therapist-led exercise program for nursing home residents with dementia is feasible, with high adherence and positive evaluations. A pilot study to refine the recruitment and intervention procedures as well as pre-and post-intervention outcome measurements is needed prior to scaling up to a larger clinical trial assessing effectiveness.

## 1 Introduction

Dementia is highly prevalent among nursing home residents, with rates between 42 % and 84 % depending on country and facility type [[1], [2], [3]]. Beyond cognitive decline, dementia is associated with reduced mobility, strength, balance, and endurance [4,5], leading to greater dependency in activities of daily living (ADL) [6] and an increased risk of falls [7]. Physical therapy, especially exercise-focused, is commonly used to address physical impairments in this population [8,9]. Physical therapists are considered the experts on exercise therapy, as endorsed by both physical therapists’ professional organizations [10] and by patients [11]. However, exercise interventions vary widely in content and supervision, limiting their comparability [[12], [13], [14], [15], [16]].

A recent review of physical therapist-supervised exercise interventions for residents with dementia included six studies: four multimodal, and two aerobic-only [17]. Aerobic interventions showed some benefits but had high bias risk. Multimodal programs showed mixed effects on performance and independence. Furthermore, the variation in outcome measures and inconsistencies in observed effects limited the strength of the conclusions. With the interpretation of the results, it must also be taken into account that none of the interventions met the recommendations from international guidelines on exercise and physical activity for nursing home residents [18] and older adults [19,20], both suggesting the incorporation of aerobic, strength, flexibility and balance exercises, with a minimum duration of 20 min and frequency of 3 times per week. This raises questions about their therapeutic validity [21].

Low adherence to exercise sessions is another concern. A meta-analysis found an average 62.3 % adherence rate among residents with dementia [22]. Some high-quality programs achieved higher rates (73–75 %) [23,24] but overall, suboptimal adherence may limit effectiveness. Exergaming, combining physical and cognitive stimulation through interactive games [25], shows promise in improving engagement [26]. An example is the use of stationary bicycles with digital video images of the environment such as *Bikelabyrinth*^Ⓡ^. A recent qualitative study [27] explored residents’ preferences, revealing a preference for professional oversight, though not necessarily by a physical therapist at all times, provided safety and quality were maintained. Building on these findings, a future intervention could incorporate a flexible supervision model, combining professional supervision with trained non-professional support.

In summary, while therapist-supervised exercise can improve function in residents with dementia, most studies fall short of guideline standards, and adherence strategies like exergaming remain underexplored. A well-designed, individualized intervention incorporating exergaming and flexible supervision may reveal its full potential, however first its feasibility must be assessed. Therefore, this study aimed to assess the feasibility (assessment completion, session adherence, adverse events, recruitment, participant appraisal, and supervisor experiences) of a 10-week physical therapist-supervised exercise intervention for nursing home residents with dementia.

## 2 Methods

### 2.1 Study design and objectives

**Fig. 1:**
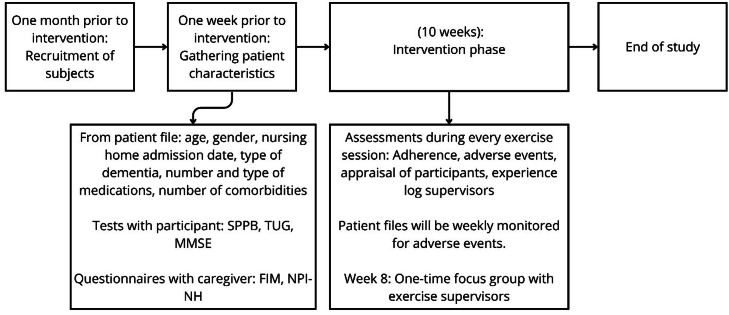
Study flow including recruitment, study length and study procedures.

We conducted a single-arm, observational study at two sites of a nursing home care organisation. The study methodology was developed in accordance with the extended Consolidated Standards of Reporting Trials (CONSORT) criteria for feasibility studies [28]. The study planning, duration and sample size was based on two previous feasibility studies involving exercise interventions for older adults with dementia [29,30], and is depicted in Fig. 1. The primary feasibility outcomes were: administration and completion of assessment instruments, with an a priori threshold of >80 %; adherence to the intervention, with a threshold of >75 %; and absence of serious adverse events attributable to the intervention. The secondary feasibility outcomes were: participant recruitment, participants’ appraisal of the intervention, and supervisors’ experiences. Ethical approval was obtained from the Medical Ethics Assessment Committee –Leiden Den Haag Delft (METC-LDD) [NL87232.058.24].

### 2.2 Setting and population

Participants were recruited and data was collected from two sites of a nursing home organization located in the urban area of Haarlem, the Netherlands during February June 2025.

### 2.3 Eligibility of participants

The attending physician assessed eligibility based on the following inclusion criteria:

• A confirmed medical diagnosis of dementia documented in the patient’s medical file
• Ability to walk at least 50 m without human assistance (use of walking aids permitted)
• No anticipated resistance to participation in the intervention, as judged by the attending physician

Exclusion criteria included:

• Behavioral problems likely to interfere with participation in a group intervention, as determined by the physician
• Any other factors identified by the physician that could render participation inadvisable for the resident

Throughout the study period, participants were allowed to continue receiving any treatments deemed appropriate in consultation with their attending physician.

## 3 Recruitment

### 3.1 Nursing home residents

At each ward, the attending physician compiled a list of potential participants based on the study’s eligibility criteria. The legal representatives of the eligible residents were contacted by the ward’s physical therapist via telephone. If the legal representative expressed interest and agreed to receive further information, an informational leaflet outlining the study was sent. One week after the initial contact, a follow-up call was made. Upon receipt of the signed consent form, the attending physical therapist informed the resident about the study and sought verbal assent. Once verbal assent was obtained, the resident was formally enrolled in the study by the study coordinator.

### 3.2 Supervisors

Supervising physical therapists were selectively recruited based on their affiliation with the participating locations and wards, postgraduate training in dementia care as well as their interest in the study. Supervisors attended one introductory meeting and two training sessions to prepare for the intervention. In addition, four fourth year physical therapy students (University of Applied Sciences, Leiden) were selectively recruited to assist with supervision. These students participated in two theoretical sessions and two practical training sessions and attended one expert lecture on dementia.

### 3.3 Intervention

The program was individually designed regarding its contents and dosage according to the FITT-VP principle: Frequency, Intensity, Time, Type, Volume, and Progression [31]. A detailed summary of the intervention is provided in Supplemental Table 1. Six instruments assessment instruments were administered one week prior to the start of the intervention: the Short Physical Performance Battery (SPPB) [32], Timed Up and Go test (TUG) [33], Mini-Mental State Examination [34], the Neuropsychiatric Inventory –Nursing Home version (NPI-NH) [35], and the Functional Independence Measure (FIM) [36]. The assessment results were used by the supervisors to enable personalization of the exercise sessions according to each participant’s individual needs and abilities. The results were further used for the external validity of the intervention by providing detailed information on the participant characteristics. The intervention comprised four sessions per week: two group exercise sessions and two individual exergaming sessions. Group sessions were 45 min long, held in the nursing home gym, and supervised by two physical therapists. They focused on strength, balance, and flexibility at moderate intensity, with exercises progressively adapted every two weeks. Individual exergaming sessions involved 20 min of supervised cycling on a stationary ergometer (Tigo 562, Thera Trainer^Ⓡ^, Germany) equipped with personalized virtual routes using Bikelabyrinth® software [Version: V5.4.1].

## 4 Feasibility outcomes

### 4.1 Primary outcomes

#### 4.1.1 Completion of the clinical assessments

An instrument was considered feasible if it could be administered and completed by at least 80 % of participants. This criterion was informed by the National Institute for Health and Care Research (NIHR) progression criteria [37] and the extended CONSORT guidelines for feasibility studies [28]. An attempt was considered valid if the participant understood the test procedure, even if they were physically unable to complete it. In addition, the number of attempts was recorded.

#### 4.1.2 Adherence to the intervention

Adherence was recorded dichotomously (yes/no) for session completion by the attending physical therapists using standardized exercise logs. Adherence was considered feasible when exceeding 75 %, as this corresponds to completing, on average, at least three exercise sessions per week.

#### 4.1.3 Adverse events

Adverse events were categorized into four types: 1) Minor and temporary, not requiring treatment by a physician or specialist, 2) Minor and temporary, requiring treatment by a physician or specialist, 3) Serious injury or illness, potentially life-threatening, 4) Death. All adverse events were discussed with the supervising physician. Adverse events classified under categories 3 and 4 were deemed serious adverse events and were handled in accordance to national guidelines [38]. The physician and the study coordinator categorized the adverse events and determined whether the event was unrelated, possibly related, or most likely related to the intervention. We considered the absence of serious adverse events related to the intervention to be feasible.

## 5 Secondary outcomes

### 5.1 Participant recruitment

To assess recruitment feasibility, we documented the number of residents screened, the number of eligible individuals approached, the number who consented to participate, and, when available, the reasons for non-participation.

### 5.2 Participants’ appraisal of the intervention

Participants’ appraisal of the exercise intervention was assessed at the end of each exercise session using a specifically for this study developed 5-point Likert scale. The scale is an adaptation of the End-of-Life in Dementia Satisfaction Scale (EOLD) [39] and the Smiley Face Assessment Scale [40]. The adaptation consisted of a printed page with the question: "How did you perceive the exercise session you just participated in?", answered on a 5-point Likert (1= Very unpleasant, 2=Unpleasant, 3=Neutral, 4=Pleasant, and 5=Very Pleasant). If a participant was unable to respond, a 3-point Likert scale consisting of the options Unpleasant, Neutral, and Pleasant in red, yellow, and green, respectively were provided.

### 5.3 Supervisor’s experiences and acceptance

Supervisor’s experiences regarding facilitators and barriers in the acceptance of the intervention were assessed using a mixed-methods approach:

• Logs documenting exercise observations: After each session, supervisors recorded their observations in the participant’s report form. Two persons (KS and JK) independently checked the notes and coded relevant information.
• Focus group discussion: In week 8 of the intervention, all supervisors participated in a focus group. The discussion was led by one of the physical therapy students (JK) and supervised by the study coordinator. The focus group was audiotaped with Microsoft Teams [Version 25,153.1010.3727.5483], transcribed with [TurboScribe.ai] and checked for correctness by JK and BW.

In addition to the standardized assessments, the following characteristics of the participating residents were retrieved from medical records: age, gender, date of nursing home admission, dementia subtype, number and type of comorbidities, total number of prescribed medications and medication categories. The number of medications and comorbidities was collected cumulatively, meaning that individual participants could be presented with more than one medication or more than one condition within a given category (e.g., multiple musculoskeletal disorders). An expert in geriatric medicine (SH) aided in categorizing the medical conditions and the medications used.

### 5.4 Statistical analysis

Baseline characteristics of the participating residents were summarized using standard descriptive statistics, specifically medians and interquartile ranges for continuous variables and frequencies for categorical variables. Recruitment feasibility was summarized using descriptive statistics. Adherence to the intervention and completion of evaluation parameters were calculated as the number of sessions attended or instruments completed, divided by the total number of sessions or instruments planned. Resident-reported experiences using Likert scales were analyzed using means and standard deviations. Supervisors’ experiences as documented in the participant report forms were analyzed using thematic analysis [41]. The themes that emerged from this preliminary analysis informed the development of the topic guide used in the focus group discussions. Focus group data were subsequently coded and organized into minor and subsequently major themes.

## 6 Results

### 6.1 Participant and supervisor recruitment

A total of 59 residents were screened for eligibility (Fig. 1) of whom 14 (24 %) were eligible for participation. All legal representatives agreed with participation (in 1 patient with the exception of the individual exercise sessions for logistical reasons), and 11 out of 14 (79 %) participants provided verbal assent. Table 1 presents their characteristics. Regarding the supervisors, eight were approached, of whom all eight agreed to participate and take part in the mandatory training.

**Table 1:** Characteristics of eleven nursing home residents with dementia participating in an observational study on a 10-week exercise program.

Characteristics	Median and range	Number ( %)
Age	84 (71–96)	
Gender (female)		8 (84.2 %)
Length of stay (months)	16 (2–95)	
Dementia type		
Alzheimer's disease		6 (54.5 %)
Vascular		1 (9.1 %)
Mixed Alzheimer's disease and vascular		1 (9.1 %)
Other		3 (27.3 %)
Medical conditions		
Cardiovascular disease		22
Vitamin deficiency		2
Dermatological disorder		12
Ear, Nose and Throat disorder		1
Gynaecological disorder		2
Intestinal disorder		4
Metabolic disorder		6
Musculoskeletal disorder		20
Neurological disorder (excluding dementia)		11
Ocular disorder		3
Psychiatric disorder		5
Pulmonary disorder		1
Rheumatic disorder		5
Surgical treatment		2
Urological/nephrological disorders		11
Medication		
Analgesics		11
Anticoagulations		6
Anticonvulsants		1
Antidepressants		3
Antihypertensive		12
Antipsychotics		3
Benzodiazepine		4
Cholesterol lowering agents		1
Cholinesterase inhibitor		2
Dermatological cream		2
Diuretics		4
Eye drops		2
Hormonal treatment		1
Inhalers		1
Laxative		4
Metabolic medicine		1
Nasal spray		2
Stomach acid reducer		3
Vitamin D and calcium supplement		11
Use of walking aid (yes)	3 out of 11	
Rolling walker	3 (100 %)	

Fig. 2

**Fig. 2:**
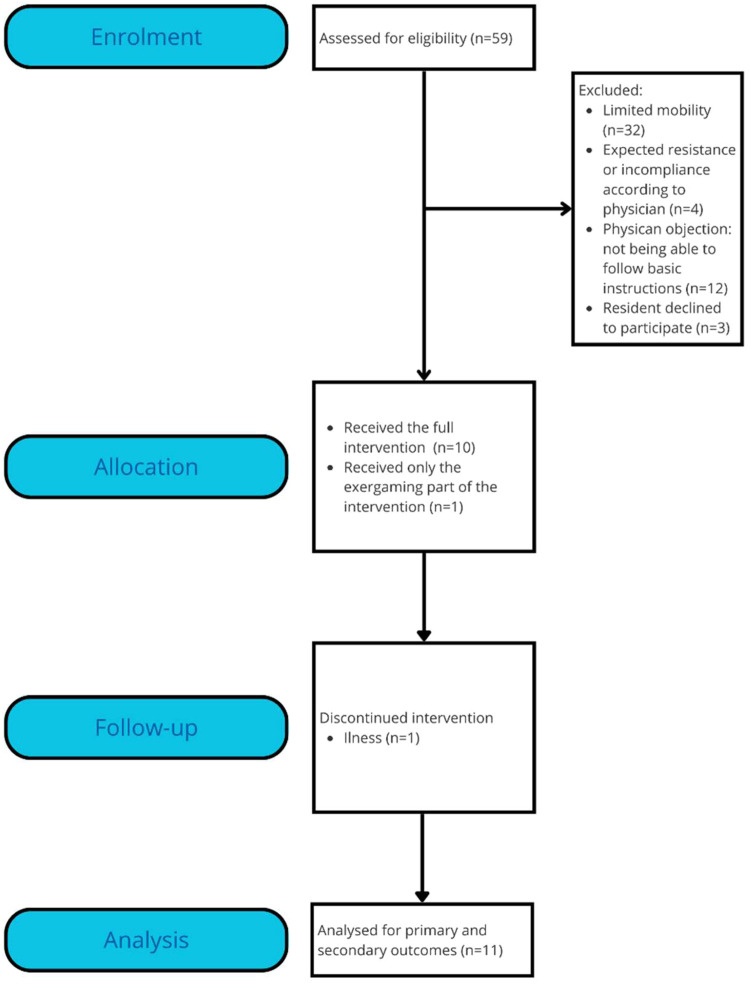
Flowchart of participant recruitment and completion of the intervention.

### 6.2 Completion of the assessment instruments

Table 2 presents the completion and outcomes of the clinical assessment instruments. Five of these, i.e. the balance test, the 4-meter walk test at a comfortable pace, the TUG test and the MMSE were completed by all 11 participants, while the FIM and the NPI were completed for all 11 participants by their professional caregiver. The SPPB was completed by 9 participants, due to the inability of 2 participants to complete the 5-times chair test component. In addition, 1 participant could not complete the 4-meter walk test at their fastest pace. Multiple attempts were needed by some participants for the balance part of the SPPB and the TUG test.

**Table 2:** Feasibility of 6 assessment instruments administered in 11 nursing home residents with dementia.

	Number of participants completing assessments	Number of attempts (median and range)	Score (median and range
Short Physical Performance Battery (range 0–12)			8 (2–12)
- Balance (range 0–4)	11	2 (1–4)	2 (0–4)
- 4 m walking speed, comfortable (meter/seconds)	11	1 (1–1)	5.9 (4.5–8.5)
- 5 times chair stand test, (seconds)	9	1 (1–3)	15.7 (10.1–20.4)
4 m walking speed, fastest (meter/seconds)	10	1 (1–2)	3.9 (2.3–7.7)
Timed Up and Go test (seconds)	11	2 (2–6)	12.7 (5.9–52.1)
Mini Mental State Examination (Range 0–30)	11	1 (1–1)	12 (7–22)
Neuropsychiatric Inventory –Nursing Home version Range (0–144)	11	1 (1–1)	7 (0–25)
Functional independence Measure (Range 18–126)	11	1 (1–1)	93 (55–107)

### 6.3 Adherence

As one participant did not engage in the combined group exercise sessions, ten residents completed the intervention. The overall adherence rate to the intervention was 89 %. Participants attended 179 of the 194 planned group exercise sessions (92 %) and 186 of the 214 planned individual exergaming sessions (87 %). Two participants had an overall adherence rate under 75 % (63 % and 71 %), and three participants completed all sessions (100 % adherence rate). Documented reasons for non-adherence included lack of willingness to participate (n = 24), medical reasons (n = 13), and participant absence from the facility at the time of the session (n = 6).

### 6.4 Adverse events

Table 3 presents the occurrence of adverse events. A total of 137 adverse events were identified, all classified as minor and temporary, with nine requiring treatment. None of these latter nine events were considered likely related to the intervention (pain caused by pressure ulcers (n = 6), nighttime falls from bed (n = 2) and depressive symptoms requiring further examination to rule out underlying causes (n = 1)). Nine of the128 adverse events that did not require treatment were classified as possibly related to the intervention, all described as fatigue or tiredness, with six occurrences reported at least one day after participation in an exercise session (details in Supplemental file 2). The highest number of reported adverse events for one resident was 21, whereas one participant had no reported adverse events.

**Table 3:** Frequency of adverse events of a 10-week exercise program for nursing home residents with dementia, categorized according to severity and with possible relation with the intervention.

	Not related to the intervention	Possibly related to the intervention	Likely related to the intervention	
CAT 1 Minor and temporary, not requiring treatment by a physician or specialist	119	9		0
CAT 2 Minor and temporary, requiring treatment by a physician or specialist	9	–		–
CAT 3 Serious injury or illness, potentially life-threatening	–	–		–
CAT 4 Death	–	–		–

### 6.5 Participants’ appraisal of the intervention

The appraisal instrument was administered after 361 of the planned 408 sessions (88 %) and was completed on 361 occasions (100 %). On 359 occasions the 5-point Likert scale was used, whereas the simplified 3-point Likert scale was used twice, due to incomprehension of the participant of the 5-point Likert scale. The median scores of the group and individual exercise sessions were 4.4 (range 1–5) and 4.3 (range 1–5), respectively, with 321 of the 359 scores (90 %) being 4 or 5 (pleasant or very pleasant) and 8 scores (2 %) being 1 or 2 (very unpleasant or unpleasant).

### 6.6 Supervisor experiences

All eight supervisors participated in the focus group discussion, resulting in four major themes (details in Supplemental File 3):

1. Motivation of residents

Supervisors noted that participants were mainly motivated by perceived health benefits and enjoyment of structured activities. Supervisors’ encouragement influenced positive attitudes, adherence, and effort levels. Effective communication was key, as residents often needed repeated explanations of the exercise purpose. A calm, reassuring approach helped foster participation.

- Supervisor 1: *“Their motivation seems to come from the general idea that being active is good for their health.”*
2. Social interaction during group exercises

Supervisors observed that social interaction had mixed effects. Watching peers exercise and receiving personal attention boosted motivation, while excessive conversation sometimes distracted participants and lowered exercise intensity. However, these distractions did not affect adherence or participants’ overall positive views of the sessions.

- Supervisor 1: *“But sometimes it’s so distracting that they just stare at each other. They laugh, and then nothing happens for like 30* s*.”*
3. Physical Environment and Setup

Participation was influenced by environmental and contextual factors. Exergaming equipment promoted engagement, while poor lighting, excessive noise, and scheduling conflicts hindered it. Support from relatives and convenient session timing further facilitated attendance and adherence.

- Supervisor 2: *“If a different activity was happening at the same time, the participants sometimes found it hard to choose between the other activity and the exercise session.”*
4. Supervisory knowledge and experience

Supervisors highlighted that dementia care expertise and adapting communication to individual needs enhanced participant motivation. A person-centered approach was deemed essential for engagement and session success.

- Supervisor 3: *“To one participant we'll say ‘we’re coming for you.’ And then you can see her smile she’ll say, ‘oh yeah.’ And right at that moment, she’s on board and comes along.”*

## 7 Discussion

This study evaluated the feasibility of a 10-week, physical therapist-supervised exercise intervention in 11 nursing home residents with dementia. Participants constituted one-quarter of screened residents. Over 80 % of the planned clinical assessments and exercise sessions were completed, and few adverse events were possibly related to the intervention, all of which were mild and transient. The intervention received a positive average rating from participants. Supervisors identified scheduling conflicts as barriers.

The clinical assessment instruments were largely feasible, consistent with prior validation in this population.^51^ Some participants required multiple attempts for the SPPB balance test [32], fastest-speed 4-m walk or TUG [33], due to apraxia, fear of falling or cognitive limitations. Taking preventive measures, such as walking alongside the participant without influencing their pace, may improve the feasibility of the fastest 4-m walk test. Regarding the TUG, positioning the physiotherapist at the turning point and providing an additional instruction to pivot, walk back to the chair, and sit down helped participants complete the TUG with fewer difficulties.

Intervention adherence exceeded that of prior meta-analyses (62.3 %) [22] and therapist-led programs (73–75 %) [23,24]. The adherence rates were not higher during exergaming than during the conventional exercise sessions, but nevertheless supervisors suggested that exergaming is acceptable to participants. While preliminary evidence suggests that exergaming may have beneficial effects on physical, cognitive, and emotional outcomes [25], whether exergaming enhances adherence or outcomes warrants further study. No serious adverse events occurred. Of all reported mild adverse events, fewer than 10 % were deemed possibly related to the intervention, mainly fatigue reported by one participant. Whether fatigue qualifies as an adverse event is debatable, as it is a common and expected response to moderate-intensity exercise [42].

The recruitment rate in this study was comparable to that reported by Toots et al.,^23^who targeted a similar population using the same selection criteria. Main barriers were logistical: insufficient residents at one site and the requirement to walk 50 m, which prevented attainment of the planned 24 participants. For a future study, expanding the screening pool (e.g., by involving additional nursing homes) would help achieve the desired sample size. Furthermore, because most intervention components can be performed with limited mobility, modifying the inclusion criteria to require only the ability to transfer from sit to stand without assistance may improve accessibility and recruitment feasibility. Most sessions were rated pleasant or very pleasant, consistent with earlier findings that residents with dementia enjoy moderate-intensity exercise [43]. The use of brief feedback tools, tailored to the cognitive abilities of this group, may warrant further development and validation.

The experiences of the intervention supervisors underscored several facilitators and barriers influencing participant acceptance of the intervention. Positive encouragement from supervisors and family members was perceived as a key facilitator, consistent with existing literature on motivation and adherence among individuals with dementia [44,45]. However, supervisors reported logistical challenges in scheduling exercise sessions without conflicting with other planned resident activities. This finding appears to contrast with previous studies, which more commonly cite a lack of available activities or infrequent family visits as limiting factors [46,47]. The reported scheduling difficulties may be specific to the Dutch long-term care context.

A strength of the study design was that feasibility was systematically assessed across multiple domains, including recruitment, adherence, safety, acceptability, and implementation, allowing for well-founded recommendations for the design of a future larger-scale trial. A limitation of the study was that it involved a relatively small sample size. Therefore, the results must be interpreted with caution. Moreover, changes over time or could not be ascertained and no estimations of effect sizes to be used in power calculations could be made that could possibly be used as outcome measures in a future clinical trial.

Although the intervention required considerable time investment and effort from both the residents and nursing home staff, the associated burden was limited and considered proportionate to the knowledge gained. The absence of serious adverse events, combined with the majority of sessions being appraised as pleasant or very pleasant by the participants, suggests that the intervention was experienced more as an enjoyable activity than as a burden. In addition, the supervisors were trained in dementia care in accordance with the study protocol, which not only benefited the intervention itself, but was also beneficial for the supervisors in their broader clinical practice. To the best of our knowledge, this is also the first study to meet the recommendations from international guidelines on exercise and physical activity for nursing home residents [18] and older adults [19,20]. Together, these findings support the rationale for the conduct of a pilot study including pre- and post-intervention outcome assessments, to allow estimation of change and variability to calculated the sample size and further justifying the required investment of time and effort from staff.

## 8 Conclusion

This study showed that recruitment for a 10-week physical therapist-led exercise program for nursing home residents with dementia was challenging, but the intervention proved to be feasible with respect to execution, adherence, safety and satisfaction. Supervisors suggested various factors affecting adherence and exercise intensity. All of these insights are valuable to improve recruitment, supervision, and personalization in future research evaluating the cost-effectiveness and long-term benefits of the intervention.

## CRediT authorship contribution statement

**Dennis Boer:** Writing – original draft, Validation, Resources, Project administration, Methodology, Investigation, Formal analysis, Data curation, Conceptualization. **Bente Winkler:** Writing – original draft, Validation, Methodology. **Charlotte Schmidt:** Writing – review & editing, Supervision, Methodology, Conceptualization. **Shanty Sterke:** Writing – review & editing, Supervision, Methodology, Conceptualization. **Wilco Achterberg:** Writing – review & editing, Supervision, Methodology, Investigation, Conceptualization. **Thea Vliet Vlieland:** Writing – review & editing, Supervision, Project administration, Methodology, Formal analysis, Data curation, Conceptualization.

## Declaration of competing interest

The authors declare that they have no known competing financial interests or personal relationships that could have appeared to influence the work reported in this paper.

## Appendix Supplementary materials

Image, application 1

Image, application 2

Image, application 3
