# Supplementary material for: A 10-week physical therapist-supervised exercise program for nursing home residents with dementia: a single arm, observational feasibility study
Source: JAR Life. 2025 Nov 3;14:100043. doi: 10.1016/j.jarlif.2025.100043 (PMC12677101; doi:10.1016/j.jarlif.2025.100043)
Supplement: Supplementary file 1 [file mmc1.docx]

## Supplemental table 1: Summary of a 10 week physical therapist supervised exercise intervention for nursing home residents with dementia, in accordance with the TiDier Template

| **TIDieR (Template for Intervention Description and Replication) checklist describing a 10-week physiotherapist-supervised exercise program for nursing home residents with dementia** | |
| --- | --- |
| 1. **Brief name** | 10-week physiotherapist-supervised exercise program for nursing home residents with dementia |
| 1. **Why** | The prevalence of dementia in nursing homes is high, ranging from 42% to 84%, and is associated with not only cognitive decline but also reduced physical capabilities such as mobility, strength, and balance, increasing the risk of falls and loss of independence. Physiotherapy, particularly exercise-based interventions, is commonly used to address these impairments, with physiotherapists seen as the key experts in this field. However, systematic reviews on exercise interventions in this population are difficult to interpret due to variation in intervention content and supervising professionals, with many studies showing inconsistent results and high risk of bias. Furthermore, most interventions fail to meet international exercise guidelines in terms of frequency, duration, and content. Adherence to exercise remains a challenge, with average rates around 62%,^1^ limiting the effectiveness of interventions. Exergaming, which combines physical and cognitive engagement in an interactive environment, shows promise in improving motivation and adherence among people with dementia. Therefore, a well-structured, guideline-based, and personalized physiotherapy intervention that includes exergaming should first be tested in a feasibility study to evaluate its practicality and potential effectiveness before proceeding to larger trials. |
| 1. **What (Materials)** | *Intervention protocol: A* detailed intervention protocol was developed in collaboration with the participating supervisors. The content of the intervention protocol was based on expert exercise guidelines, ^2,3^ a recent systematic review on physiotherapy for nursing home residents with dementia, ^4^ two studies exploring the needs and preferences of nursing home residents and their family caregivers regarding physiotherapy and exercise, ^5,6^ as well as clinical experience with this population.  In order to maintain the contrast in the study, participating physical therapists were instructed not to disseminate or show the protocol or its contents to others by any means.  *Treatment registration forms:* physical patient log forms were used to record the contents and evaluation of every treatment session, including the registration of possible (serious) adverse effects.  *Materials used in the group exercise sessions:* weighted vest up to 20kg were used to increase the load of the standing and sitting exercises. Ankle weights were used to increase the load of the leg extension and the steps exercises. A balance pad and small materials such as sand bags, colored rings and colored weights were used in the dynamic balance exercises. A pully system with a maximum load of 24 kg was used for the back-strength exercises.  *Materials used in the individual exergaming sessions:*  The individual exergaming sessions consisted of aerobic cycling using a seated ergometer bicycle (Tigo 562, Thera Trainer©, Germany). The stationary bicycle was equipped with Bikelabyrinth® video software [Version: V5.4.1], which displayed pre-recorded cycling routes through various locations on a television screen. |
| 1. **What (Procedures)** | **Clinical assessments**  *Physical functioning*  Lower extremity muscle strength, static balance, and walking speed were assessed using the Short Physical Performance Battery (SPPB)^7^ comprising a balance test (scored 0–4 points) involving standing in side-by-side, semi-tandem, and tandem positions; a gait speed test (0–4 points) measured over a 4-meter walk; and a chair stand test (0–4 points) in which the participant rises from and sits down on a chair five times. Higher scores indicate better physical performance. In addition to the 4-meter walk at a comfortable pace, a 4-meter walk at the fastest safe pace was conducted to provide additional information to support the personalization of the exercise sessions. Mobility was assessed using the Timed Up and Go (TUG) test,^8^ which measures the time (in seconds) a participant takes to rise from a standard armchair, walk three meters, turn around, return, and sit down again. The TUG was conducted twice, with the average of both attempts used.  *Cognition*  Cognition was evaluated using the Dutch version of the Mini-Mental State Examination (MMSE)^9^ consisting of 11 open-ended questions or tasks. It evaluates various cognitive functions, including attention and orientation, memory, registration, recall, calculation, language, and praxis. The scores range from 0 to 30, with lower scores indicating lower cognitive levels.  *Neuropsychiatric symptoms of dementia*  Neuropsychiatric symptoms of dementia were assessed with the Neuropsychiatric Inventory – Nursing Home version (NPI-NH)^10^ including the frequency and severity of neuropsychiatric symptoms of a resident in the previous week as observed by the nursing staff. It addresses ten behavioral areas and two types of neurovegetative changes: delusions; hallucinations; agitation/aggression; depression/dysphoria; anxiety; euphoria/elation; apathy/indifference; disinhibition; irritability/lability; aberrant motor behavior; sleep and nighttime behavior disorders; appetite and eating disorders. The scores of each behavioral area range from 0 to 12, where 0 indicates absence, and 12 represents the maximum frequency and severity level. The total score on the NPI ranges from 0 to 144 with higher scores indicating the presence of frequent and severe neuropsychiatric symptoms.  *Independence in ADL*  Independence in ADL was assessed using the Functional Independence Measure (FIM).^11^ It consists of 18 items, covering areas such as self-care, mobility, communication, and social cognition. Each item is scored on a scale from 1 to 7, reflecting the level of assistance required. The total FIM score ranges from 18 to 126, with higher scores indicating greater independence.  ***Group Exercises***  *Type:* Muscle strengthening exercises (using own weight or additional weights); Neuromotor (functional) exercises (motor skills such as balance, flexibility or coordination)  *Dosage:* Duration of a treatment session was 45 minutes, with the desired moderate intensity. The intensity was increased progressively based on supervisors perception.  ***Individual exergaming exercises***  *Type:* Aerobic exercises in the form of cycling on a seated bike.  *Dosage:* Duration of a treatment session was 20 minutes. Participants cycled on their preferred intensity, but were stimulated to cycle with a moderate intensity. The intensity was evaluated biweekly.  ***Evaluation of adverse events and appraisal of the intervention***  Every session, participants were actively asked about adverse events, and the their appraisal of the intervention was evaluated. |
| 1. **Who provided** | *Group exercises:* Each group exercise session included a maximum of six residents and was supervised by a physiotherapist and a movement therapist. Two physiotherapists and two movement therapists with experience and expertise of exercise for nursing home residents with dementia were purposively recruited. Supervisors attended one introductory meeting and two training sessions to prepare for the intervention.  *Individual exergaming exercises:* four fourth year physiotherapy students (University of Applied Sciences, Leiden) were selectively recruited to assist with supervision of the exergaming component of the intervention (see: Intervention). These students participated in two theoretical sessions and two practical training sessions, and attended one expert lecture focused on dementia knowledge. Prior to the start of the study, they completed a qualification assessment, supervised by the study coordinator, to ensure their competency to supervise the intervention. |
| 1. **How** | All sessions were on location and face-to-face |
| 1. **Where** | Group exercise sessions were conducted in the nursing home gymnasium. To ensure privacy while preserving a familiar and accessible environment, the individual exergaming area was situated in a secluded yet central part of the nursing home. |
| 1. **When, and how much** | The intervention comprised four sessions per week: two supervised group exercise sessions and two individual exergaming sessions. The total duration was 10 weeks. |
| 1. **Tailoring** | Physical therapists were instructed to observe signs of fatigue or exertion. Residents were asked about their perceived exertion, and exercises were adjusted (made more of less challenging) depending on the perceived exertion. Objective measures of exertion such as the BORG^12^ scale were deemed not feasible in this population. |
| 1. **Modifications** | The intervention was not modified |
| 1. **How well (planned)** | Adherence to the intervention was calculated by dividing the number of sessions attended by the total number of sessions offered, and multiplying the result by 100 to obtain a percentage. In addition, the duration of participation in each session, expressed in minutes, was recorded to provide further insight into adherence. Adherence of the supervisors to the intervention protocol was monitored by conducting bi-weekly calls from the study coordinator with the supervisors. |
| 1. **How well (actual)** | The overall adherence rate to the intervention was 89%. Adherence to the individual exergaming sessions was 87%, while adherence to the group exercise sessions was 92%. Four participants completed all scheduled sessions, achieving 100% adherence. The lowest individual adherence rate observed was 62.5%. Documented reasons for non-adherence included lack of willingness to participate (6% of sessions), medical reasons (1%), and participant absence from the facility at the time of the session (1%). |
| 1. Di Lorito C, Bosco A, Booth V, Goldberg S, Harwood RH. Adherence to exercise interventions in older people with mild cognitive impairment and dementia : A systematic review and meta-analysis. *Prev Med Rep*. 2020;19(June):101139. doi:10.1016/j.pmedr.2020.101139  2. Izquierdo M, de Souto Barreto P, Arai H, et al. Global consensus on optimal exercise recommendations for enhancing healthy longevity in older adults (ICFSR). *Journal of Nutrition, Health and Aging*.*Elsevier B.V.* 2025;29(1). doi:10.1016/j.jnha.2024.100401  3. Souto P De, Morley JE, Chodzko-zajko W, et al. Recommendations on Physical Activity and Exercise for Older Adults Living in Long-Term Care Facilities : A Taskforce Report. *J Am Med Dir Assoc*. 2016;17(5):381-392. doi:10.1016/j.jamda.2016.01.021  4. Boer D, Schmidt C, Sterke S, Schoones J, Elbers R, Vliet Vlieland T. Characteristics and Effectiveness of Physical Therapist-Supervised Exercise Interventions for Nursing Home Residents With Dementia: A Systematic Review. Makaroun LK, ed. *Innov Aging*. 2024;8(7). doi:10.1093/geroni/igae061  5. Boer DE, Sterke S, Schmidt CB, Vliet Vlieland TPM. The perceptions, needs and preferences of informal caregivers of nursing home residents with dementia regarding physical therapy: A qualitative study. *Geriatr Nurs (Minneap)*. 2022;44:167-175. doi:10.1016/j.gerinurse.2022.01.014  6. Boer D, Nibbering R, Schmidt C, et al. Exploring the physiotherapy and exercise needs and preferences of nursing home residents with dementia: A qualitative study. *J Alzheimers Dis Rep*. 2025;9. doi:10.1177/25424823251349166  7. Guralnik JM, Simonsick EM, Ferrucci L, et al. A short physical performance battery assessing lower extremity function: association with self-reported disability and prediction of mortality and nursing home admission. *J Gerontol*. 1994;49(2):M85-M94. doi:10.1093/geronj/49.2.M85  8. Podsiadlo D, Richardson S. The Timed “Up & Go”: A Test of Basic Functional Mobility for Frail Elderly Persons. *J Am Geriatr Soc*. 1991;39(2):142-148. doi:10.1111/j.1532-5415.1991.tb01616.x  9. Kok R, Verhey F. Dutch translation of the Mini Mental State Examination. Published online 2002.  10. De Jonghe JFM, Kat MG, Kalisvaart KJ, Boelaarts L. Neuropsychiatric inventory questionnaire. *J Neuropsychiatry Clin Neurosci*. 2003;34(2):74-77.  11. Granger C V., Hamilton BB, Linacre JM, Heinemann AW, Wright BD. Performance profiles of the functional independence measure. *Am J Phys Med Rehabil*. 1993;72(2).  12. BORG GAV. Psychophysical bases of perceived exertion. *Med Sci Sports Exerc*. 1982;14(5):377-381. doi:10.1249/00005768-198205000-00012 | |
