# Supplementary material for: A 10-week physical therapist-supervised exercise program for nursing home residents with dementia: a single arm, observational feasibility study
Source: JAR Life. 2025 Nov 3;14:100043. doi: 10.1016/j.jarlif.2025.100043 (PMC12677101; doi:10.1016/j.jarlif.2025.100043)
Supplement: Supplementary file 2 [file mmc2.docx]

## Supplemental Table 2: Cumulative presentation of adverse events in a 10 week physical therapist supervised exercise intervention for nursing home residents with dementia

| CATEGORY 1: Minor and temporary, not requiring treatment by a physician or specialist. Not related to the intervention |  |
| --- | --- |
| Muscle pain | 3 |
| Pain (other) | 30 |
| Restlessness | 30 |
| Fatigue/tiredness | 4 |
| Falls | 24 |
| Feeling unwell | 2 |
| Confusion | 4 |
| Depressive symptoms | 2 |
| Agression | 6 |
| Demanding behaviour | 1 |
| Emotional | 3 |
| Suspicious behaviour | 3 |
| Irritated | 1 |
| Agitation | 2 |
| Hallucinations | 1 |
| Angry | 1 |
| Sad | 2 |
| **Total** | **119** |

| CATEGORY 1: Minor and temporary, not requiring treatment by a physician or specialist. Possibly related to the intervention |  |
| --- | --- |
| Fatigue/tiredness | 9 |

| CATEGORY 2  Minor and temporary, requiring treatment by a physician or specialist. Not related to the intervention |  |
| --- | --- |
| Pressure ulcers | 6 |
| Falls at night | 2 |
| Depressive symptoms | 1 |
| **Total** | **9** |
