# Supplementary material for: A 10-week physical therapist-supervised exercise program for nursing home residents with dementia: a single arm, observational feasibility study
Source: JAR Life. 2025 Nov 3;14:100043. doi: 10.1016/j.jarlif.2025.100043 (PMC12677101; doi:10.1016/j.jarlif.2025.100043)
Supplement: Supplementary file 3 [file mmc3.docx]

## Supplemental table 3: Four major themes resulting from thematic analysis of the focus group with 8 supervisors.

| **Theme** | **Subtheme** |
| --- | --- |
| Motivation to Participate | Beneficial for health |
|  | Enthusiasm for the activity |
|  | Planning (e.g., routine, other activities) |
|  | Role of facilitators was also mentioned, possibly relevant as separate theme |
|  | Involvement of close ones adds stimulation and memory sharing |
| Interaction | Inhibiting factors: group dynamics, quitting, etc. |
|  | Stimulating factors: seeing others exercise, preference for conversation |
| Physical Environment and Experience | Materials (e.g., beanbag, bike, route) |
|  | Space (e.g., planning, familiarity, size) |
|  | Stimuli (e.g., music, interaction, route) |
|  | Adaptations and facilitation increase feasibility |
| Facilitation | Individual approach (plan, explanations, cognition) |
|  | General interaction style in dementia |
|  | Connection and recognition |
|  | Person-dependent aspects (talkative, quiet, etc.) |
